# Trends in Cost Attributable to Kidney Transplantation Evaluation and Waiting List Management in the United States, 2012-2017

**DOI:** 10.1001/jamanetworkopen.2022.1847

**Published:** 2022-03-10

**Authors:** Xingxing S. Cheng, Jialin Han, Jennifer L. Braggs-Gresham, Philip J. Held, Stephan Busque, John P. Roberts, Jane C. Tan, John D. Scandling, Glenn M. Chertow, Avi Dor

**Affiliations:** 1 Division of Nephrology, Department of Medicine, Stanford University School of Medicine, Palo Alto, California; 2 Division of Nephrology, Department of Medicine, University of Michigan, Ann Arbor; 3 Division of Abdominal Transplantation, Department of Surgery, Stanford University School of Medicine, Palo Alto, California; 4 Division of Transplant Surgery, Department of Surgery, University of California, San Francisco; 5 George Washington University, Milken Institute School of Public Health, Washington, DC

## Abstract

**Importance:**

While recent policy reforms aim to improve access to kidney transplantation for patients with end-stage kidney disease, the cost implications of kidney waiting list expansion are not well understood. The Organ Acquisition Cost Center (OACC) is the mechanism by which Medicare reimburses kidney transplantation programs, at cost, for costs attributable to kidney transplantation evaluation and waiting list management, but these costs have not been well described to date.

**Objectives:**

To describe temporal trends in mean OACC costs per kidney transplantation and to identify factors most associated with cost.

**Design, Setting, and Participants:**

This economic evaluation included all kidney transplantation waiting list candidates and recipients in the United States from 2012 to 2017. A population-based study of cost center reports was conducted using data from all Center of Medicare & Medicaid–certified transplantation hospitals. Data analysis was conducted from June to August 2021.

**Exposures:**

Year, local price index, transplantation and waiting list volume of transplantation program, and comorbidity burden.

**Main Outcomes and Measures:**

Mean OACC costs per kidney transplantation.

**Results:**

In 1335 hospital-years from 2012 through 2017, Medicare’s share of OACC costs increased from $0.95 billion in 2012 to $1.32 billion in 2017 (3.7% of total Medicare End-Stage Renal Disease program expenditure). Median (IQR) OACC costs per transplantation increased from $81 000 ($66 000 to $103 000) in 2012 to $100 000 ($82 000 to $125 000) in 2017. Kidney organ procurement costs contributed to 36% of mean OACC costs per transplantation throughout the study period. During the study period, transplantation hospitals experienced increases in kidney waiting list volume, kidney waiting list active volume, kidney transplantation volume, and comorbidity burden. For a median-sized transplantation program, mean OACC costs per transplantation decreased with more transplants (−$3500 [95% CI, −$4300 to −$2700] per 10 transplants; *P* < .001) and increased with year ($4400 [95% CI, $3500 to $5300] per year; *P* < .001), local price index ($1900 [95% CI, $200 to $3700] per 10-point increase; *P* = .03), patients listed active on the waiting list ($3100 [95% CI, $1700 to $4600] per 100 patients; *P* < .001), and patients on the waiting list with high comorbidities ($1500 [9% CI, $600 to $2500] per 1% increase in proportion of waitlisted patients with the highest comorbidity score; *P* = .002).

**Conclusions and Relevance:**

In this study, OACC costs increased at 4% per year from 2012 to 2017 and were not solely attributable to the cost of organ procurement. Expanding the waiting list will likely contribute to further increases in the mean OACC costs per transplantation and substantially increase Medicare liability.

## Key Points

**Question:**

How much do evaluation and waiting list management cost before kidney transplantation?

**Findings:**

In this economic evaluation of cost reports from all certified transplant hospitals in the United States, kidney transplantation–related Organ Acquisition Cost Center (OACC) payments from Medicare amounted to $1.32 billion in 2017 (3.7% of total Medicare End-Stage Renal Disease Program expenditure), and OACC cost per transplantation increased from $81 000 in 2012 to $100 000 in 2017. Transplantation waiting list size and comorbidities were associated with an increase in OACC cost per transplant.

**Meaning:**

These findings suggest that pre–kidney transplantation cost is increasing rapidly and these increases may be accelerated by efforts to expand waiting list access.

## Teaser

This economic evaluation describes temporal trends in mean Organ Acquisition Cost Center costs per kidney transplantation and identifies the factors most associated with cost.

## Introduction

Kidney transplantation is the preferred treatment for end-stage kidney disease (ESKD).^1^ Because of the expenses associated with the alternative, (ie, maintenance dialysis), kidney transplantation has been frequently lauded as a cost-saving treatment. Held et al^2^ estimate the benefit of each kidney transplantation at approximately $1.1 million, using a cost-benefit analysis framework. However, most patients with ESKD in the United States are not treated with kidney transplantation.

The largest health insurance provider in the United States is the federally funded Medicare program. Medicare eligibility is conferred by age 65 years or older, permanent disability, and, uniquely, ESKD. Given the high costs of maintenance dialysis and the burden of hospitalizations and other complications, the ESKD Program represents 7.2% of overall Medicare fee-for-service expenditures as of 2018.^3^

Medicare payment for kidney transplantation occurs via 2 mechanisms. The transplant procedure itself and posttransplant care, including physician visits and hospitalizations, are included in the traditional fee-for-service model, in which Medicare prospectively sets a payment rate for each service. However, much of pretransplant evaluation and maintenance specific to transplantation, ie, health services done for the purpose of transplantation and excluding maintenance dialysis and other standard-of-care treatments for patients with ESKD, are funded via the Organ Acquisition Cost Center (OACC) mechanism.^4^ Each fiscal year, Medicare-certified transplantation hospitals report all allowable costs attributable to pretransplant care and organ procurement as OACC on the Medicare Cost Report, and Medicare subsequently reimburses the transplantation hospital the Medicare portion of the OACC cost. The Medicare ratio is calculated as follows:

(No. of Medicare primary beneficiaries who received a kidney transplant + No. of kidneys procured) / (No. of kidney transplants + No. of kidneys procured).

OACC cost includes the costs of evaluating and maintaining potential recipients on the waiting list, evaluating living donors, living donor nephrectomy and donor aftercare, and procuring deceased donor kidneys from organ procurement organizations (OPOs) that levy a standard acquisition charge per kidney that represents the cost of procuring and transporting the organ.^5^ From 2013 through 2017, the mean standard acquisition charge, or cost of procuring the organ, was $36 000 per kidney.^5^ One would expect the mean OACC cost per kidney transplantation over the same period to be higher, as it not only includes the standard acquisition charge but also the other costs described previously.^5^ In 2000, OACC costs per kidney transplantation already ranged from $23 000 to $60 000, with a median of $38 000.^4^ In contrast, the US Renal Data System, the primary reporting system for outcomes and costs related to ESKD care in the US,^3^ has estimated the entire OACC cost as $25 000 per kidney transplantation, from 2000 until the latest version of the Annual Data Report in 2020; this low figure has been used in most of the literature examining costs in kidney transplantation.

A reexamination of the recent trends in, and determinants of, OACC costs is warranted. First, given increasing costs in most aspects of health care and the complete absence of downward pressure on OACC (ie, reimbursement at cost), we would expect OACC costs to increase at least as fast, if not faster, than other health care services. Second, recent efforts to promote kidney transplantation in the United States include attempts to increase patients’ access to the waiting list, eg, the addition of the percentage of patients waitlisted as a performance metric for dialysis facilities^6^ and considerations of an opt-out system in which all patients with ESKD are referred for kidney transplantation by default.^7^ Understanding the OACC, in conjunction with all the other costs of kidney transplantation, will enable a better projection of the costs that might be incurred by these changes in policy.

Here, we aim to report trends in the mean OACC cost per kidney transplantation in the US and specifically Medicare’s share of total OACC. We also examine the determinants of OACC to identify factors most associated with the cost increase.

## Methods

### Data

The research in this paper was approved by the Stanford institutional review board, and the need for informed consent waived given that the study did not use patient-level data. We followed the Consolidated Health Economic Evaluation Reporting Standards (CHEERS) reporting guideline.

The Centers for Medicare & Medicaid Services (CMS) mandates that each CMS-certified hospital file a CMS-2552-10 (Hospital Cost Report) annually. Transplantation-related costs are included in worksheet D4 (OACC) and form the basis for the CMS settlement paid to transplantation hospitals. We obtained available CMS-2552-10 data for fiscal years 2012 to 2017 from the National Bureau of Economic Research, which included all US transplantation hospitals.^8^ The fiscal year varied from hospital to hospital. We extracted data pertaining to kidney transplantation, including total OACC cost incurred by the kidney transplantation program (line 61, column 1); the Medicare ratio (line 64, column 2); net Medicare acquisition cost or the dollar amount for which Medicare is responsible (line 69, column 1); and the number and dispositions of organs procured and transplanted at the program (lines 70-84). We supplemented hospital cost report data with the following: (1) measures of transplantation program waiting list and transplantation volume and comorbidity burden, obtained from the Scientific Registry of Transplant Recipients^9^; (2) local organ procurement cost (ie, standard acquisition charge) based on the affiliated OPO, based on OPO cost report forms (available for 2013-2017 only)^10^; and (3) data on local cost of living^11^ (eAppendix 1 in the Supplement).

Patients on the waiting list can be status active, meaning they can receive organ offers at any time, or status inactive, meaning they are not ready for transplantation and will not be receiving offers. Given wide variabilities in the proportion of patients listed as active vs inactive at each center, we estimated waiting list size separately by both the total waiting list volume (the total number of patients listed) and the waiting list active volume (the total number of patients listed as status active) on January 1 of each calendar year. Comorbidity burden was represented by the distribution of the Estimated Post-Transplant Survival (EPTS) scores in patients on waiting lists and those who have received transplants: for each patient, EPTS is calculated from age, diabetes status, dialysis vintage, and prior transplantation and ranges from 1 to 100; the EPTS score is inversely proportional to the patient’s comorbidity burden and projected posttransplantation survival.^12^

### Statistical Analysis

Primary outcomes were (1) OACC cost per kidney transplantation and (2) OACC cost per kidney transplantation attributable to costs other than deceased donor organ procurement, calculated as OACC cost per kidney transplantation minus the standard acquisition charge set by the primary OPO of the transplant hospital. Results are reported as median (IQR) in the text. Results are reported in US dollars and not inflation adjusted, given our goal of describing temporal trends.

To examine the association of transplantation program characteristics with the primary outcomes, we used a generalized estimating equation, incorporating an unstructured covariance matrix to account for association within the same transplantation program across different years. We modeled the outcome as the natural log of cost to account for the skewed distribution of cost and to derive valid estimates with regression modeling. Given the small size of the coefficients, we modeled annual transplantation and waiting list volume (total and active) in 10s and 100s, respectively. For instance, 16 transplants would be 1.6, and 16 patients on the waiting list would be 0.16. We also modeled transplant and waiting list volumes as a linear and a quadratic term, given their distributions, and compared performance of linear vs quadratic variable structures by examining the quasilikelihood under independence of each model.

We conducted statistical analyses using SAS version 9.4 (SAS Institute). Statistical significance was set at *P* < .05, and all tests were 2-tailed.

## Results

Our final study set consisted of 1335 hospital-years from 2012 through 2017, of which 930 hospital-years from 2013 through 2017 were linked to OPO cost data. Table 1 displays baseline characteristics. During the study period, transplantation hospitals had increases in median (IQR) kidney waiting list volume (2012-2013: 273 [110-569] patients; 2016-2017: 281 [128-599] patients; *P* < .001), median (IQR) kidney waiting list active volume (2012-2013: 167 [65-346] patients; 2016-2017: 172 [74-369] patients; *P* < .001), and median (IQR) kidney transplant volume (2012-2013: 52 [24-94]; 2016-2017: 60 [28-117]; *P* < .001). The increase in transplant volume was mostly associated with an increase in deceased donor transplant volume (median [IQR] deceased donors, 2012-2013: 35 [15-65]; 2016-2017, 44 [18-83]; *P* < .001). The increase in transplant volume was seen in all United Network of Organ Sharing (UNOS) regions except region 10 (Midwest), where it remained relatively flat (eAppendix 2 in the Supplement). Comorbidities, as estimated by EPTS, also increased during the study period, in both patients on the waiting list and patients receiving transplantation, with a shift in the proportion of patients with the lowest comorbidity burden (EPTS 0-20) to patients with higher comorbidity burden. The Medicare ratio stayed constant at 0.72 throughout the study period, meaning that Medicare was liable for approximately 72% of total OACC costs throughout the study period. The standard acquisition charge per kidney also increased over the study period by approximately $1000, or 3%, per year (from a median [IQR] of $33 000 [$30 000-$35 000] in 2012-2013 to $35 000 [$32 000-$38 000] in 2016-2017), and contributed to 36% of the mean OACC cost per kidney transplantation throughout the study period.

**Table 1.** Baseline Characteristics of All Transplantation Hospitals in the United States Performing Kidney Transplants, 2012-2017

Characteristic	Median (IQR) by period, No.			*P* value^a^
	2012-2013	2014-2015	2016-2017	
Unique transplantation hospitals, No.	227	221	221	.95
Waiting list volume^b^	273 (110-569)	293 (116-614)	281 (128-599)	<.001
Waiting list active volume^b^	167 (65-346)	171 (69-352)	172 (74-369)	<.001
Transplant volume^c^	52 (24-94)	53 (27-96)	60 (28-117)	<.001
Living donor transplants	15 (6-31)	13 (5-30)	14 (6-31)	.77
Deceased donor transplants	35 (15-65)	37 (17-69)	44 (18-83)	<.001
Patients on waiting list in each risk category, %				
EPTS 0-20	31 (27-37)	30 (25-35)	29 (25-34)	<.001
EPTS 21-40	31 (27-33)	30 (27-33)	30 (27-32)	
EPTS 41-60	21 (18-23)	21 (18-23)	22 (19-23)	
EPTS 61-80	12 (9-14)	12 (9-15)	13 (10-15)	
EPTS 81-100	5 (2-7)	5 (3-7)	5 (3-7)	
Patients receiving transplantation in each risk category, No.				
EPTS 0-20	36 (31-42)	35 (30-42)	36 (30-43)	.01
EPTS 21-40	27 (22-30)	27 (22-31)	26 (21-30)	
EPTS 41-60	19 (14-23)	19 (15-22)	19 (15-22)	
EPTS 61-80	10 (6-13)	12 (8-15)	12 (8-15)	
EPTS 81-100	4 (2-6)	4 (2-6)	4 (2-7)	
Mean Medicare ratio	0.72 (0.61-0.82)	0.73 (0.62-0.82)	0.72 (0.61-0.81)	.67
Local price index	151 (138-181)	150 (137-181)	151 (137-181)	.81
Local standard acquisition charge for kidney, thousands of $^d^	33 (30-35)	34 (31-37)	35 (32-38)	<.001

Abbreviation: EPTS, Estimated Post Transplant Survival.

^a^ *P* value is calculated for trend over year (continuous variable) using a generalized estimating equation.

^b^ Waiting list volume indicates the total number of patients on the waiting list on day 1 of the fiscal year; waiting list active volume indicates the total active patients on the waiting list on day 1 of the fiscal year.

^c^ Transplant volume was calculated as the number of transplants during the fiscal year.

^d^ Local standard acquisition charge for kidney is the standard acquisition charge at the organ procurement organization in the same donor service area as the transplant hospital. Only available for 2013 to 2017.

**Figure 1.**
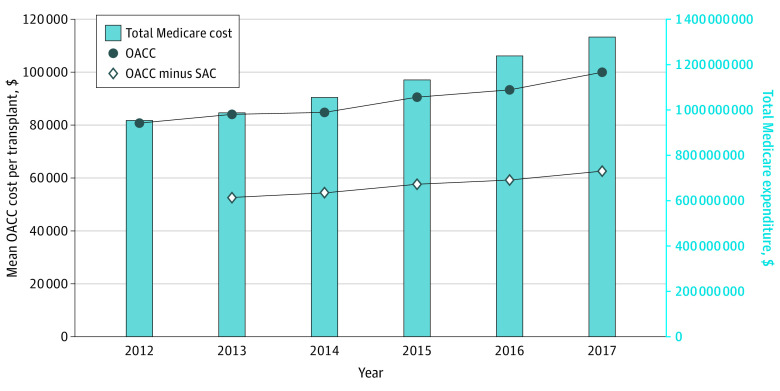
Indirect Costs of Kidney Transplantation Over Time OACC indicates Organ Acquisition Cost Center; SAC, standard acquisition cost.

**Figure 2.**
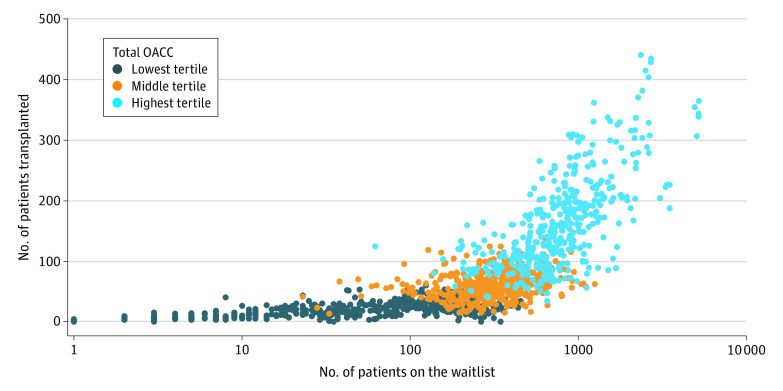
Association of Transplantation and Waiting List Volumes With Total Organ Acquisition Cost Center (OACC) Costs at the Program-Year Level

Medicare share of OACC costs increased from $0.95 billion in 2012 to $1.32 billion in 2017 (Figure 1). Median (IQR) OACC costs per kidney transplantation increased from $81 000 ($66 000-$103 000) per kidney transplantation in 2012 to $100 000 ($82 000-$125 000) per kidney transplantation in 2017. Median (IQR) OACC costs attributable to costs other than organ procurement (OACC less SAC) increased from $53 000 ($36 000-$81 000) per kidney transplantation in 2013 to $62 000 ($46 000-$86 000) per kidney transplantation in 2017. OACC per kidney transplantation varied by UNOS region, with the highest costs in regions 9 (New York, Vermont), 4 (Texas), and 5 (Southwest). Estimates for most regions did not achieve statistical significance (eAppendix 2 in the Supplement). Total OACC costs (at the program-year level) were positively associated with both transplant volume and waiting list volume (Figure 2). The lowest tertile ranged from $0 to $3 600 000; middle tertile, $3 700 000 to $8 300 000; highest tertile, $8 300 000 to $39 000 000.

**Figure 3.**
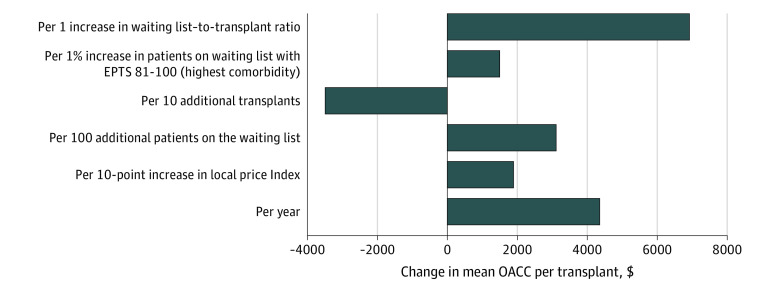
Association of Year, Price Index, and Transplantation Program Characteristics With the Mean Organ Acquisition Cost Center (OACC) Cost per Transplant The estimates are for a median US transplant program in 2017 (local price index, 151; 172 active patients on the waiting list; 60 transplants performed; and 5% of patients on the waiting list with high comorbidity burden).

We chose models in which transplant and waiting list volumes were represented by linear terms. eAppendix 3 in the Supplement presents comparison of the models with different covariate structures. Mean OACC costs per kidney transplantation were associated with a decrease as transplantation programs performed more transplants and an increase with year, local price index, more patients listed active on the waiting list, and more patients on the waiting list with EPTS of 81 to 100 (denoting higher comorbidity on the waiting list) (Table 2). The pattern was similar when we examined OACC costs attributable to costs other than organ procurement (OACC less SAC) (Table 2). Translated to a transplant program in 2017 with median local price index, transplantation and waiting list volumes, and comorbidity burden, mean OACC costs per kidney transplantation were associated with a decrease of $3500 (95% CI, $2700-$4300) per 10 transplants performed (*P* < .001) and an increase with year ($4400 [95% CI, $3500-$5300] per year; *P* < .001), local price index ($1900 [95% CI, $200-$3700] per 10-point increase; *P* = .03), more patients listed active on the waiting list ($3200 [95% CI, $1700-$4600] per 100 patients; *P* < .001), and more patients on the waiting list with high comorbidities ($1500 [95% CI, $600-$2500] per 1% increase in proportion of waitlisted patients with the highest comorbidity score; *P* = .002) (Figure 3).

**Table 2.** Univariate and Multivariate Model Illustrating the Association Between Transplantation Hospital Characteristics and Mean OACC Cost per Kidney Transplantation OACC Costs Attributable to Items Other Than Organ Procurement Costs^a^

Factor	Univariate model (unadjusted)		Multivariate model (adjusted)	
	Effect size estimate, % (95% CI)	*P* value	Effect size estimate, % (95% CI)	*P* value
**OACC cost per kidney transplantation**				
Year, per year	3.6 (2.6 to 4.4)	<.001	4.4 (3.5 to 5.3)	<.001
Local price index, per 10 points	1.3 (−0.4 to 3.0)	.12	1.9 (0.2 to 3.7)	.03
Waiting list volume, per 100 patients	−0.3 (−0.8 to 0.4)	.64	NA	NA
Waiting list active volume, per 100 patients	0.7 (−1.5 to 0.2)	.12	3.2 (1.7 to 4.6)	<.001
Transplant volume, per 10 transplants	−2.1 (−2.7 to −1.5)	<.001	−3.5 (−4.3 to −2.7)	<.001
Percentage of patients on waiting list with EPTS 81-100, per 1%	1.4 (0.4 to 2.4)	.008	1.5 (0.6 to 2.5)	.002
Percentage of patients receiving transplantation with EPTS 81-100, per 1%	0.3 (−0.3 to 0.8)	.40	NA	NA
**OACC cost minus standard acquisition charge per kidney transplantation**				
Year, per year	2.9 (0.8 to 5.1)	.007	4.6 (2.6 to 6.7)	<.001
Local price index, per 10 points	−0.6 (−3.4 to 2.4)	.72	NA	NA
Waiting list volume, per 100 patients	−0.7 (−1.6 to 0.2)	.11	4.2 (2.1 to 6.2)	<.001
Waiting list active volume, per 100 patients	−0.1 (−0.3 to −0.1)	.04	NA	NA
Transplant volume, per 10 transplants	−2.9 (−3.7 to −2.0)	<.001	−5.4 (−6.7 to −4.1)	<.001
Percentage of patients on waiting list with EPTS 81-100, per 1%	2.1 (0.2 to 3.7)	.03	2.6 (1.0 to 4.2)	<.001
Percentage of patients receiving transplantation with EPTS 81-100, per 1%	0.2 (−1.1 to 0.7)	.63	NA	NA

Abbreviations: EPTS, estimated post-transplant survival; NA, not applicable; OACC, Organ Acquisition Cost Center.

^a^ Both outcomes were transformed as a log function. All variables with *P* < .20 in the univariate model were included in the multivariate model. Association with cost is the translation of the median estimate in the multivariate model into percentage change.

In a companion analysis, we looked at the waiting list–to-transplant ratio, or the ratio between the number of patients on the waiting list to the number of patients receiving transplantation in each program-year, and its association with mean OACC cost per kidney transplantation. The median waiting list–to-transplant ratio was 4.7 (IQR, 3.0-7.1; range, 0-97). The higher the ratio, the higher the mean OACC cost per kidney transplantation (eAppendix 4 in the Supplement). An increase from a waiting list–to-transplant ratio of 1 (from a median of 5 to 6 patients on the waiting list per patients receiving transplantation) was associated with an $6930 increase (after adjustment for year, price index, and waiting list comorbidity) in mean OACC cost per kidney transplantation (Figure 3).

## Discussion

In this study, we described the national trends in OACC costs related to kidney transplantation, both as total Medicare liability and on a per-kidney transplantation basis, from fiscal years 2012 through 2017, in the United States. We found that 36% of OACC costs was attributable to deceased donor organ procurement costs, and that larger waiting list size, more complex waiting list case-mix, and a higher waiting list–to-transplant ratio were associated with higher mean OACC cost per kidney transplantation.

From a quantitative standpoint, Medicare’s liability of OACC costs ($1.32 billion in 2017) represents 3.7% of total Medicare spending on the ESKD program ($36 billion in 2017).^3^ In contrast, total Medicare spending on surgical care delivery decreased from 2011 through 2014.^13^ Median OACC costs per kidney transplantation of $100 000 in 2017, not the $25 000 as assumed by the USRDS cost reports, would increase the per-person expenditure of the first year of kidney transplantation from $133 000 to $208 000. The increase in median OACC costs per kidney transplantation of 4.4% contrasts with Medicare’s expenditure per patient with ESKD (increasing by 2.6% over the same study period).^3^ These numbers illustrate the extent to which the OACC system supports kidney transplantation programs in the United States. Prior costing studies, both single-center^14^ and multicenter,^15^ have shown that the prospective payment rates set by Medicare for transplantation episodes via the Diagnosis-Related Group (DRG) are frequently insufficient to cover the expenses of kidney transplantation, especially when the transplantation uses nonstandard kidneys or more complex immunologic protocols.^16^ OACC, in its retrospective, at-cost reimbursement structure, represents a way for kidney transplantation programs to maintain fiscal viability, but it is also not subject to cost pressures the same way prospectively reimbursed services are.

By combining the OACC cost database with an organ procurement cost database (which provides the standard acquisition charge per kidney at each transplantation program), we were able to show that only one-third of OACC costs was attributable to organ procurement. The rest represents the cost of evaluating potential kidney transplantation recipients and managing an ever-growing kidney transplantation waiting list. Due to the shortage of transplantable kidneys, many patients on the kidney transplantation waiting list die or are removed before a kidney becomes available. They incur costs during the evaluation and maintenance process on the waiting list but accrue no benefit of kidney transplantation other than the possible, albeit unsubstantiated, benefit of more engagement in their care while being on the waiting list. The costs of patients on a waiting list who do not receive a transplant are shifted to the OACC, the bulk of which Medicare pays based on the proportion of Medicare recipients who do receive transplantation. It is therefore unsurprising that the OACC cost per kidney transplantation increases with measures of waiting list size, waiting list case-mix, and the waiting list–to-transplant ratio and decreases with actual transplants performed. These findings highlight the importance of efficiency—or how quickly transplantation programs aid a patient in moving from evaluation to waiting list to the actual transplant—as a key factor in OACC cost. More than 10 years ago, Abecassis^17^ drew the analogy between efficiency in transplantation program operations and the efficiency in inventory management by manufacturers. Our study provides the empirical data to support the claim that to contain OACC costs, transplantation programs need to examine the efficiency of their practices. Similarly, public and payment policy should be aligned with the purpose of enhancing efficiency.

Our finding has major implications for the recent policies to increase kidney transplantation referral and waitlisting. Access to the waiting list is a process, rather than an outcome, measure: it is not an end in itself. However, the fact that a waiting list even exists is evidence that the shortage of kidneys, not a shortage of patients eligible for transplantation, is limiting the volume of kidney transplantation in the United States. Measures solely to increase waiting list access, unaccompanied by measures to improve organ availability, are unlikely to succeed in increasing the number of kidney transplants. Our findings suggest that such measures may also have the unintended consequence of diminishing efficiency and substantially increasing the OACC and overall costs of the Medicare ESKD program. Under the current reimbursement system, the OACC is uniquely shielded from cost containment measures that are built into prospective payment systems, such as DRG-based reimbursement or the ESKD Prospective Payment System. Even if transplantation programs were to wish to introduce measures to reduce OACC costs, eg, deferring work-up and waitlisting until patients without living donors have had enough dialysis time to be within reach of a deceased donor transplant, such practices act in the opposite direction of recent policy changes to increase waiting list access and would likely be resisted by referrers. Blanket policies to increase waiting list access without increasing organ availability could not only overwhelm transplantation program workforces but also threaten the solvency of the entire Medicare ESKD program.

### Limitations

This study has limitations. Two main limitations exist regarding the data source. First, as a measure of the costs of pretransplant care, ie, care above and beyond routine care that a patient with ESKD would only receive given the possibility of a transplant, the OACC underestimates the true cost. Consider, for instance, the case of a potential recipient with no cardiac symptoms who undergoes a screening test for ischemic heart disease as a part of kidney transplantation evaluation, as is standard practice^18^ and supported by society guidelines.^19^ The costs of the initial stress test may be assigned to the OACC, but follow-up care, including repeated testing, coronary angiogram, revascularization, and treatment of any complication that arises, becomes the standard of care for a patient and is billed to the patient’s insurance. Given the number of screening tests recommended for kidney transplantation evaluation^20^ that are not otherwise indicated in asymptomatic patients with ESKD, eg, cardiac testing and routine cancer screening,^21^ any tests assigned to OACC could substantially increase the total cost of care for that patient, costs that would not be otherwise incurred had there been no transplant evaluation. Second, substantial variability exists in how transplantation programs assign costs to OACC and report them on the Hospital Cost Report.^4^ For instance, a patient who needs to undergo a stress test for a kidney transplantation evaluation may choose to undergo testing at a local facility, which bills it to the patient’s insurance directly, thereby bypassing the OACC mechanism completely. This is a second way in which the OACC underestimates the true cost of pretransplant care. The Hospital Cost Report also does not breakdown reported costs by indication or phase of care, eg, donor evaluation, transplant candidate evaluation, or waiting list maintenance. It is therefore impossible to directly deduce what each transplantation-specific service costs. Part of our future direction is to apply a cost function approach, as has been applied to dialysis modalities^22^ and organ procurement costs,^23^ to estimate the costs of each transplant-specific service.

## Conclusions

Our work points to the financial impact of OACC to transplantation hospitals and to the Medicare ESKD program and helps to explain the rapid increase in cost. It also highlights the importance of understanding the relative value propositions in the management of ESKD. High-value efforts include forestalling ESKD and, when that fails, increasing living and deceased donation, enhancing deceased donor kidney usage, and increasing the lifespan of transplanted kidneys to decrease the need for retransplantation.^24^ The value of pretransplant screening practices need to be rigorously evaluated in light of their cost implications. As one of the most successful of all modern medical interventions, the value of a kidney transplantation cannot be overstated, but the relative values of services and policies in its run-up phase need to be better defined.

## Notes

Supplement. **eAppendix 1. **Data Sources **eAppendix 2. **Associations of UNOS Region With Transplantation Volumes and Mean OACC per Kidney Transplantation **eAppendix 3.** Alternative Parameterization of Transplantation and Waiting List Volumes as a Linear Term and a Quadratic Term **eAppendix 4. **Companion Analysis: Association of Waiting List–to-Transplantation Ratio With Mean OACC Cost per Kidney Transplantation
