# Supplementary material for: Trends in Cost Attributable to Kidney Transplantation Evaluation and Waiting List Management in the United States, 2012-2017
Source: JAMA Netw Open. 2022 Mar 10;5(3):e221847. doi: 10.1001/jamanetworkopen.2022.1847 (PMC8914579; doi:10.1001/jamanetworkopen.2022.1847)
Supplement: Supplement. — eAppendix 1. Data Sources eAppendix 2. Associations of UNOS Region With Transplantation Volumes and Mean OACC per Kidney Transplantation eAppendix 3. Alternative Parameterization of Transplantation and Waiting List Volumes as a Linear Term and a Quadratic Term eAppendix 4. Companion Analysis: Association of Waiting List–to-Transplantation Ratio With Mean OACC Cost per Kidney Transplantation [file jamanetwopen-e221847-s001.pdf]

Supplemental Online Content

Cheng XS, Han J, Braggs-Gresham JL, et al. Trends in cost attributable to kidney transplantation evaluation and waiting list management in the United States, 2012-2017. *JAMA Netw Open.* 2022;5(3):e221847. doi:10.1001/jamanetworkopen.2022.1847

eAppendix 1. Data Sources

eAppendix 2. Associations of UNOS Region on Transplantation Volumes and Mean OACC per Kidney Transplantation

eAppendix 3. Alternative Parameterization of Transplantation and Waiting List Volumes as a Linear Term and a Quadratic Term

eAppendix 4. Companion Analysis: Association of Waiting List–to-Transplantation Ratio on Mean OACC Cost per Kidney Transplantation

This supplemental material has been provided by the authors to give readers additional information about their work.

© 2022 Cheng XS et al. *JAMA Network Open.*

eAppendix 1. Data Sources

Figure S1. Data sources used. Grey circles represent the data sources and boxes represent the data elements taken from each data source.

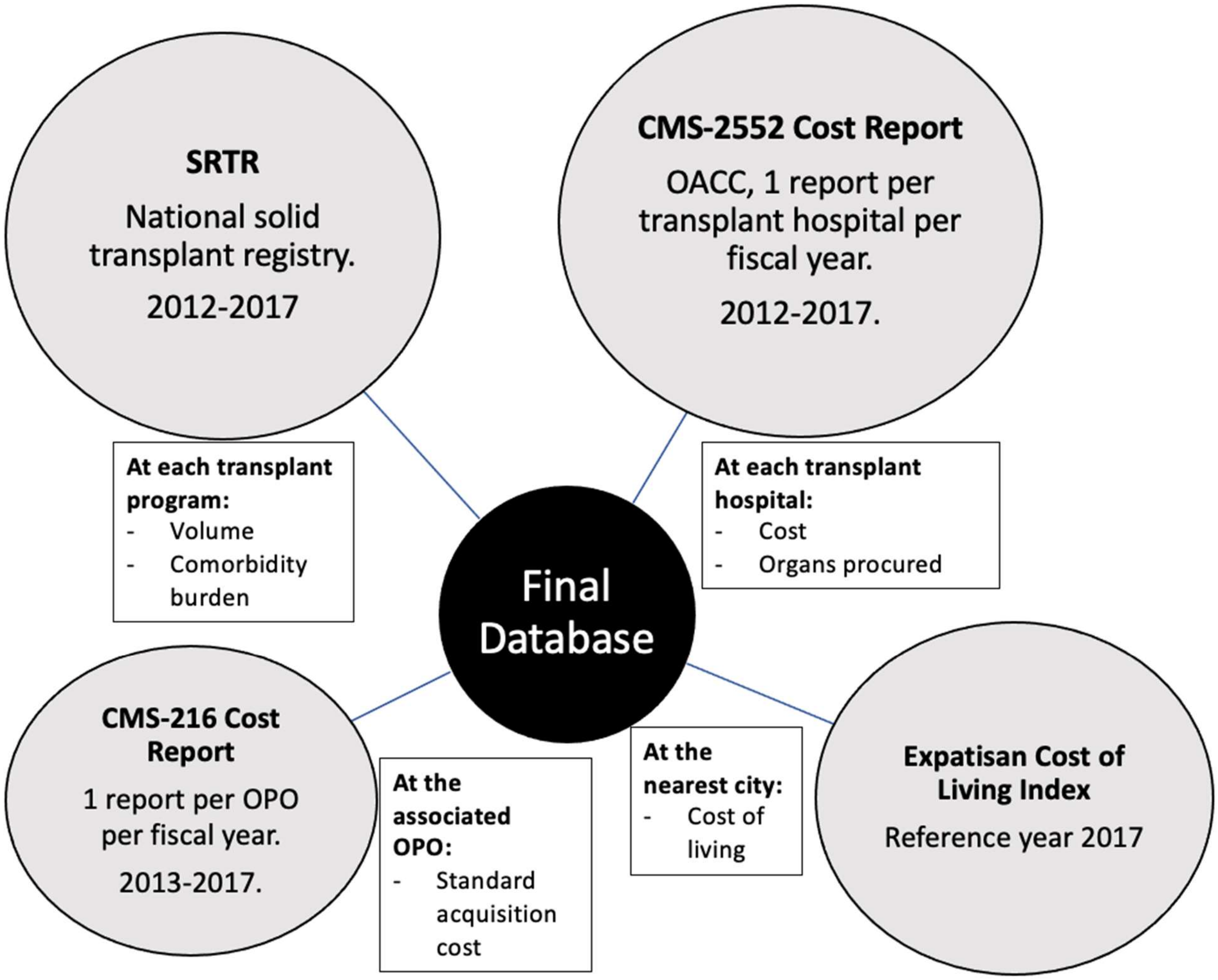

© 2022 Cheng XS et al. *JAMA Network Open.*

eAppendix 2. Associations of UNOS Region on Transplantation Volumes and Mean OACC per Kidney Transplantation

Figure S1. Transplant volumes across the study period, stratified by UNOS Region.

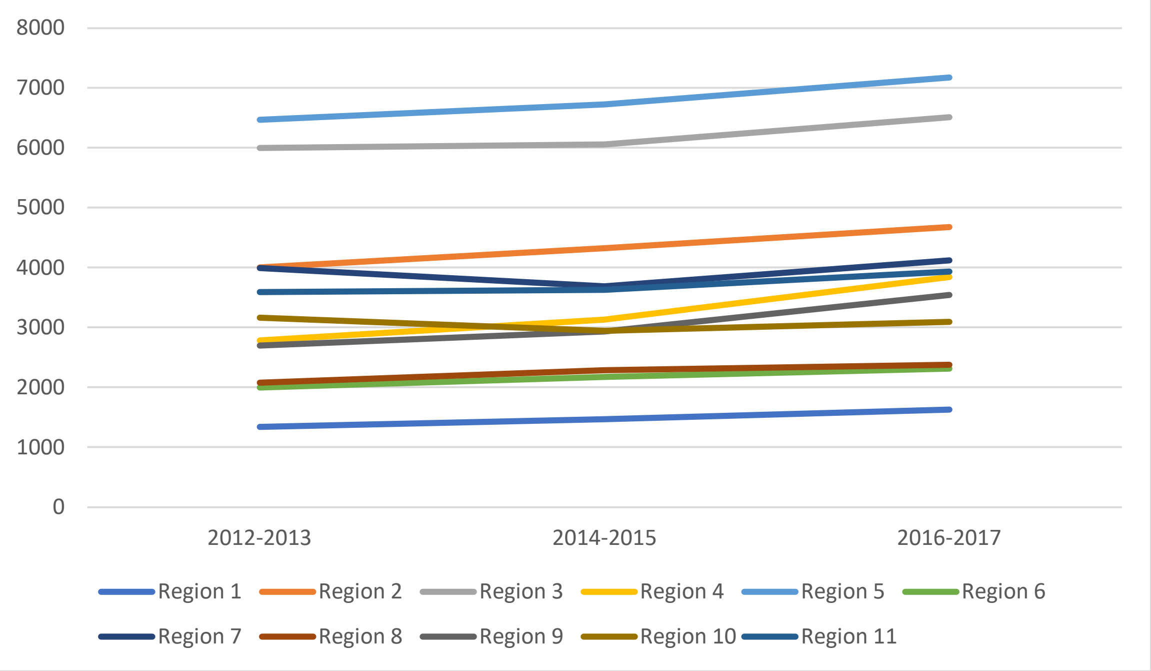

Figure S2. Effects of UNOS Region on mean OACC per kidney transplant. Univariate model only contains region in the model. Multivariate model includes price index as well as region.

	Univariate Model		*Multivariate Model*	
Region	*Effect Size*	*p-value*	*Effect Size*	*p-value*
1	-4.8%	0.60	-7.5%	0.43
2	+15%	0.16	+9.6%	0.36
3	+2.3%	0.80	-0.1%	1.00
4	+22%	0.07	+17%	0.15
5	+18%	0.09	+15%	0.16
6	+7.4%	0.59	+4.1%	0.77
7	+10%	0.30	+8.7%	0.38
8	-17%	0.07	-17%	0.06
9	+29%	0.03	+14%	0.23
10	-0.2%	0.98	-0.4%	0.97
11	ref	n/a	ref	n/a

© 2022 Cheng XS et al. *JAMA Network Open.*

eAppendix 3. Alternative Parameterization of Transplantation and Waiting List Volumes as a Linear Term and a Quadratic Term

Table 2 in the paper illustrate the main results illustrating the relationship between 1) OACC per KTx and 2) OACC less Standard Acquisition Charge per KTx. We modelled transplant and waitlist volumes as a linear term.

We also explored alternative models in which we modelled the transplant and waitlist volumes as quadratic terms. To compare model fit, we examined the quasilikelihood of independence model criterion (QIC) of each model under different variable parameterization assumptions:

Outcome	Transplant and Waitlist Volumes as Linear Terms	Transplant and Waitlist Volumes as Quadratic Terms	Better Model
Mean OACC per KTx	QIC=1327	QIC=1326	Linear
Mean OACC less Standard Acquisition Charge per KTx	QIC=920	QIC=919	Linear

The following table illustrates the results of the models in which transplant and waitlist volumes are modelled as quadratic terms.

**Outcome: OACC per KTx**					
	**Univariate Model (Unadjusted)**		**Multivariate Model (Adjusted)**		
	**Estimate (95% CI)**	**p-value**	**Estimate (95% CI)**	**Effect on Outcome**	**p-value**
Year (per year)	0.035 (0.026, 0.043)	<0.0001	0.045 (0.037, 0.052)	+4.6% (3.58 5.3)	<0.0001
Local price index (per 10)	0.013 (-0.004, 0.030)	0.1	0.021 (0.003, 0.038)	+2.1% (0.3, 3.9)	0.02
Waitlist volume (per 100)	0.0023 (-0.011, 0.016)	0.7	-	-	-
Waitlist volume (per 100) squared	-0.0002	0.3	-	-	-

© 2022 Cheng XS et al. *JAMA Network Open.*

	(-0.0005, 0.0001)				
Waitlist active volume (per 100)	-0.0056 (-0.0249, 0.0136)	0.6	-	-	-
Waitlist volume (per 100) squared	-0.0001 (-0.0007, 0.0005)	0.9	-	-	-
Transplant volume (per 10)	-0.061 (-0.077, -0.045)	<0.0001	-0.069 (-0.083, -0.055)	na	<0.0001
Transplant volume (per 10) squared	0.0014 (0.0009, 0.0018)	<0.0001	0.0014 (0.001, 0.002)	na	<0.0001
Percent waitlisted patients with EPTS 81-100 (per 1%)	0.014 (0.004, 0.024)	0.008	0.016 (0.007, 0.026)	+1.6% (0.7, 2.6)	0.007
Percent transplanted patients with EPTS 81-100 (per 1%)	0.0027 (-0.0026, 0.0080)	0.4	-	-	-
**Outcome: OACC minus SAC**					
	**Univariate Model (Unadjusted)**		**Multivariate Model (Adjusted)**		
	**Estimate (95% CI)**	**p-value**	**Estimate (95% CI)**	**Effect on Outcome**	**p-value**
Year (per year)	0.029 (0.008, 0.050)	0.007	0.045 (0.026, 0.065)	+4.6% (2.6, 6.7)	<0.0001
Local price index (per 10)	-0.006 (-0.035, 0.024)	0.7	-	-	-
Waitlist volume (per 100)	-0.0030 (-0.024, 0.018)	0.8	-	-	-
Waitlist volume (per 100) squared	-0.0001 (-0.0006, 0.0003)	0.6	-	-	-
Waitlist active volume (per 100)	-0.0148 (-0.0441, 0.0146)	0.3	-	-	-
Waitlist volume (per 100) squared	0.0001 (-0.0008, 0.0009)	0.9	-	-	-
Transplant volume (per 10)	-0.074 (-0.097, -0.052)	<0.0001	-0.086 (-0.108, -0.064)	na	<0.0001
Transplant volume (per 10) squared	0.0016	<0.0001	0.002 (0.001, 0.002)	na	<0.0001

© 2022 Cheng XS et al. *JAMA Network Open.*

	(0.0009, 0.0022)				
Percent waitlisted patients with EPTS 81-100 (per 1%)	0.014 (0.004, 0.024)	0.008	0.032 (0.017, 0.048)	+3.3% (1.7, 4.9)	0.007
Percent transplanted patients with EPTS 81-100 (per 1%)	0.0027 (-0.0026, 0.0080)	0.4	-	-	-

© 2022 Cheng XS et al. *JAMA Network Open.*

eAppendix 4. Companion Analysis: Association of Waiting List–to-Transplantation Ratio on Mean OACC Cost per Kidney Transplantation

Waitlist-to-transplant ratio was log-transformed, since the variable was heavily right-skewed (median 4.7, IQR 3.0-7.1, range 0-97).

	**Univariate Model (Unadjusted)**		**Multivariate Model (Adjusted)**	
	**Effect Size Estimate (95% CI)**	**p-value**	**Effect Size Estimate (95% CI)**	**p-value**
Year (per year)	+3.6% (2.6, 4.4)	<0.0001	+3.9% (3.1, 4.7)	<0.0001
Local price index (per 10)	+1.3% (-0.4, 3.0)	0.12	-0.1% (-1.6, 1.4)	0.87
Percent waitlisted patients with EPTS 81-100 (per 1%)	+1.4% (0.4, 2.4)	0.008	-0.8% (-1.6, 0.04)	0.06
Log (Waitlist-to-transplant ratio)	+31% (25, 37)	<0.0001	+31% (25, 38)	<0.0001

© 2022 Cheng XS et al. *JAMA Network Open.*
